# Aqueous synthesis of functionalized copper sulfide quantum dots as near-infrared luminescent probes for detection of Hg^2+^, Ag^+^ and Au^3+^

**DOI:** 10.1038/s41598-017-10904-y

**Published:** 2017-09-13

**Authors:** Weilin Du, Lei Liao, Li Yang, Aimiao Qin, Aihui Liang

**Affiliations:** 1 Key Lab New Processing Technology for Nonferrous Metals & Materials Ministry of Education, Guangxi Key Laboratory in Universities of Clean Metallurgy and Comprehensive Utilization for Non-ferrous Metals Resources, College of Materials science & engineering, Guilin University of Technology, Guilin, China; 2 College of Environment science & engineering, Guilin University of Technology, Guilin, China; 3 College of Environment & Resource, Guangxi Normal University, Guilin, China

## Abstract

Stable water-soluble copper sulfide(Cu_2_S) quantum dots(QDs) with near-infrared emission were synthesized using N-acetyl-L-cysteine(NAC) as a modifier in aqueous solution and nitrogen atmosphere at room temperature. The product was characterized by TEM, XRD, XPS, FT-IR, FL and UV-VIS spectrometers. Effects of preparation conditions such as pH values, the molar ratio of reactants, temperature, and metal ions on the fluorescence properties of Cu_2_S QDs were discussed. Under optimal conditions, the prepared Cu_2_S QDs with average diameter about 2–5 nm show a near-infrared emission at 770 nm with the excitation wavelength of 466 nm, and have a good detection sensitivity for ions of Hg^2+^, Ag^+^ and Au^3+^, based on the characteristic of fluorescence quenching. The fluorescence quenching mechanism was proposed via electron transfer with cation exchange, which based on the theory of Hard-Soft-Acid-Base (HSAB) and Ksp value of metal–sulfide.

## Introduction

Quantum dots(QDs) have been extensively applied in electronic devices^1^ and solar cell^2^, particularly in the recent years, many researchers committed to synthesize QDs with properties of water-solubility, narrow emission spectra and high fluorescence intensity and low toxicity, served as biomarkers^3^ in biology and medicine and a fluorescent probe to detect heavy metal in analysis^4, 5^. Due to their high stability, low toxicity and unique properties, copper(I) sulfide(Cu_2_S) nanoparticles(NPs) are attracting increasing attention from researchers. For example, Zhou and co-works successfully synthesized 2D Cu_2_S nanosheets, using vulcanized polyisoprene as the precursor of sulfur. The prepared Cu_2_S nanosheets could act as effective cocatalysts for photocatalytic hydrogen production^6^. Xin Liu *et al*. prepared highly self-doped Cu_2−x_E(E = S, Se) nanocrystals through by a varying reaction time and coordinating solvent composition to control size of Cu_2−x_S, ranging from 2.8 to 13.5 nm^7^. Yue Wu *et al*.^8^ successfully obtained a hexagonal structure of Cu_2_S nanocrystals with near-infrared emission in a mixed anhydrous solvent of dodecanethiol and oleic acid at a temperature higher 100 °C. The Cu_2_S nanocrystals were demonstrated their application as an active light absorbing component in combination with CdS nanorods to make a solution - processed solar cell. Previous reports show that surface functionalization of QDs is one of the most important aspects of designing and preparing the desired QDs for intended optical and biomedical applications. It is proved that different stabilizers can make Cu_2_S produce different optical properties^9^. However, to the best of our knowledge, a little detailed information is available on an aqueous synthetic method for stable near-infrared (NIR) emitting Cu_2_S QDs with N-acetyl-L-cysteine(NAC) as capping ligand at room temperature.

Heavy metal ions such as Hg^2+^, Ag^+^ and Au^3+^, etc. have severe effects on the human health and environment even at very low concentration level^10, 11^. Therefore, development of a highly selective and sensitive sensor to monitor heavy metal ions is of great important and interesting^12, 13^. Fluorescence sensors are one of the useful techniques for the detection of heavy metal ions. Nowadays, nanocrystalline semiconductors or QDs attract great attention from chemists as a new class of the inorganic fluorophores due to the unique superior properties than the classical organic fluorophores. CdS QDs with different capping molecules are one of the most popular QDs used as metal ion sensor probes. Recently, several types of QDs used as a sensor probe for detection of heavy metal ions also have been reported^14, 15^. However, most of the reported existing QDs-based sensors were emitting at wavelengths below 550 nm. Thus far, only a few NIR-emitting QDs-based sensors have been reported.

In order to increase the detection sensitivity and selectivity, many researchers committed to study of the detection mechanism of QDs. Thitima Khantaw *et al*. reported that free Ag^+^ in silver nanoparticles can enhance the fluorescence intensity of Cys–CdS QDs by formation of Ag–SR complex absorbed on the surface of the CdS QDs, which can create more radiative centers and block nonradiative e^−^/h^+^ recombination^16^. Kexin Zhang and co-workers also reported that L-Cys capped CdS:Eu QDs were used as a fluorescence probe to detect Hg^2+^ ions, the fluorescence quenching mechanism was due to the facilitating non-radiative e^−^/h^+^ recombination through an effective electron transfer process between functional groups on the surface of QDs and Hg^2+^ ^17^. Jing Wang group synthesized NAC capped CdTe/CdS@ZnS–SiO_2_ NIR-emitting QDs for detection Hg^2+^ which were based on the electron transfer^18^. And Baojuan Wang *et al*. came up with the decrease luminescence due to formation of aggregates of GQDs induced by Hg^2+^ ^11^.

From intensive reviews with available resources, there has been no report focusing on both the synthesis and the determination of heavy metal ions by the near-infrared emission fluorescent Cu_2_S QDs. In this work, stable water-soluble Cu_2_S QDs with NIR emission were firstly synthesized in aqueous solution at room temperature with NAC as a modifier. The one-step synthesis of the NAC capped Cu_2_S QDs is also firstly served as a fluorescent probe for detecting heavy metal ions such as Hg^2+^, Ag^+^, Au^3+^, and etc. Based on the characteristic of fluorescence quenching of NAC capped Cu_2_S QDs by the heavy metal ions, detection mechanisms are discussed.

## Methods

### Materials

NAC, Na_2_HPO_4_, KH_2_PO_4_, CaCl_2_ were purchased from Sinopharm Chemical Reagent limited Co., Ltd. Na_2_S∙9H_2_O, NaOH, NH_4_Cl, BaCl_2_, KCl, MnCl_2_, NaCl, Al_2_(SO_4_)_3_∙18H_2_O, CdCl_2_, FeCl_3_, AgNO_3_ were purchased from Xilong Chemical Industry Co., Ltd. CuCl_2_∙2H_2_O was purchased from Nanzhao Xinghua Chemical Factory. Na_2_CO_3_ was obtained from Guangdong chemical Reagent Engineering-technological Research and Development Center. HAuCl_4_∙4H_2_O was obtained from Shanghai Tuosi Co. Ltd. Al(NO_3_)_3_∙9H_2_O was obtained from Tianjin GUANGFU Fine Chemical Research Institute. HgCl_2_ was purchased from Tongren Guizhou chemical reagent Factory. NAC was of Biological reagent grade and other chemicals were of analytical reagent grade, all reagents were used without further purification. All the solutions were prepared in three levels of purified water.

### Apparatus

All fluorescence measurements were acquired on a VARIAN fluorescence spectrometer which was recorded by 10 excitation slits, 10 emission slits, voltage of 700 V. The transmission electron microscopy (TEM) images and high- resolution TEM images of the prepared Cu_2_S QDs were carried out on JEM-2100F. X-ray diffraction (XRD) patterns were collected on X’Pert PRO X-ray diffractometer with Cu Kα radiation. FT-IR spectra were measured with a Thermo Nexus 470 FT-IR Fourier Transform Infrared Spectroscopy to identify some characteristic functional groups. X-ray photoelectron spectroscopy (XPS) were recorded by ESCALAB 250Xi photoelectron spectroscopy. UV–visible spectra and absorbance measurements were recorded on an UV/Vis/NIR spectrophotometer (Shimadzu UV-3600). Particle size was also obtained with dynamic light scattering (DLS) using particle size analyzer (Malvern Zetasizer Nano ZS 90, U.K.).

### Synthesis of NAC capped Cu_2_S QDs

Under light shielding conditions, 0.0979 g of NAC was dissolved into 100 mL purified water in three-necked flask. The CuCl_2_ solution (2 mL 0.1 mol/L) was added under vigorous stirring for 30 seconds and produced a turbid liquid. The 1 mol/L NaOH solution was added dropwise with constant stirring to the turbid liquid become clear. Then Na_2_S solution (1 mL 0.1 mol/L) was injected into the mixed solution under strong stirring, immediately the solution turned orange. After stirring for one minute, the NAC capped Cu_2_S QDs were obtained, the pH value of the system was 7.0. The whole experiment process was bubbling with nitrogen. The prepared Cu_2_S QDs were sealed and stored in dark at room temperature. The powder sample of Cu_2_S QDs was gained by vacuum freeze drying.

### Measurement procedures

In this work, different pH values of phosphate buffer solution(PBS) were made by mixing a different volume of 0.05 mol/L Na_2_HPO_4_ solution and 0.05 mol/L KH_2_PO_4_ solution.

For determination of heavy metal ions, the following procedure was carried out. In a 10 mL volumetric tube, 1.2 mL NAC capped Cu_2_S QDs(1 × 10^−3^ mol/L) solution, certain amounts of Hg^2+^ and other metal ions were sequentially added. The mixture was diluted to 3 mL with 0.05 mol/L PBS(pH 6.98) solution and equilibrated for 20 min. The fluorescence intensity was measured at λ_em_/λ_ex_ = 770/466 nm. The fluorescence intensity of NAC–Cu_2_S QDs was assigned as F_0_. The fluorescence intensity after adding heavy metal ions was assigned as F. The change value of the fluorescence intensity (ΔF = F − F_0_) was plotted versus the concentration of heavy metal ions to obtain a calibration curve.

## Results and Discussion

### Characterization of NAC capped Cu_2_S QDs

**Figure 1:**
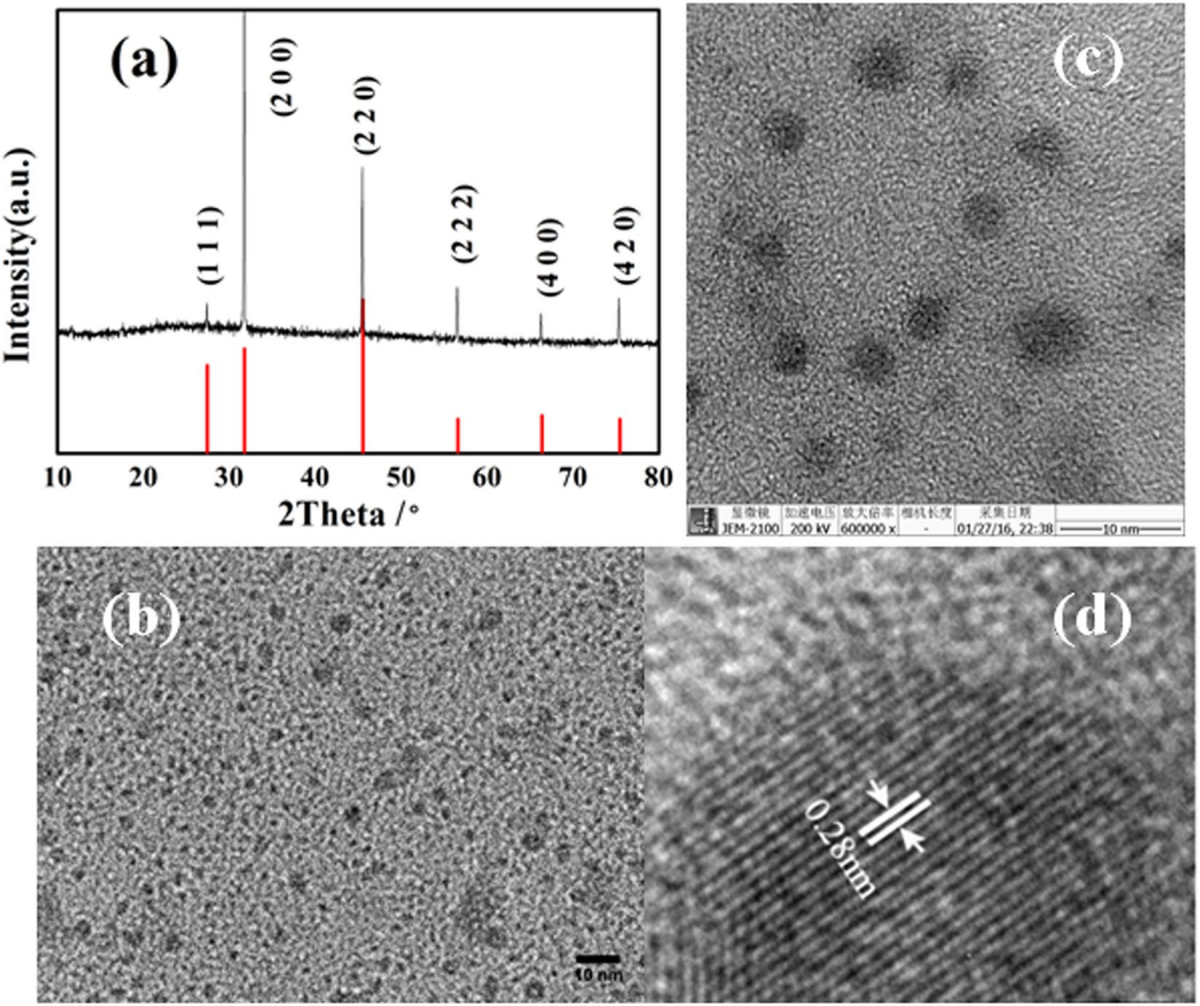
XRD pattern (**a**), TEM (**b**),(**c**) and HRTEM (**d**) images of the as-synthesized Cu_2_S QDs.

XRD pattern of as-prepared powder sample is shown in Fig. 1(a). XRD pattern shows that the entire diffraction pattern matches well with the standard data of the cubic Cu_2_S phase (PDF 01-084-1770). We tracked the reaction process, firstly, NAC was dissolved into water, then CuCl_2_ solution was added, the solution immediately changed from dark brown to white precipitate, indicating that Cu^2+^ was reduced to Cu^+^ and formation of stable NAC-Cu^+^ complexes^19, 20^. After NaOH solution added, the solution becomes clear, which indicates that the NAC-Cu^+^ complexes were gradually dissolved in solution and decomposed free Cu^+^ ions, in the present of active S^2−^ ions, reaction between Cu^+^ and S^2−^ ions occurs to form the stable Cu_2_S.

TEM and HRTEM images of as-prepared Cu_2_S QDs are shown in Fig. 1(b–d). From the TEM images Fig. 1(b and c), it is obvious that the particles were well mono-dispersed, featuring spherical shape with the size ranging from 2 nm to 5 nm. HRTEM image (Fig. 1d) displays that the QDs are highly crystalline and the regular spacing of the clear lattice planes is calculated to be 0.28 nm, which is in good agreement with the spacing of (2 0 0) crystallographic plane of cubic Cu_2_S.

**Figure 2:**
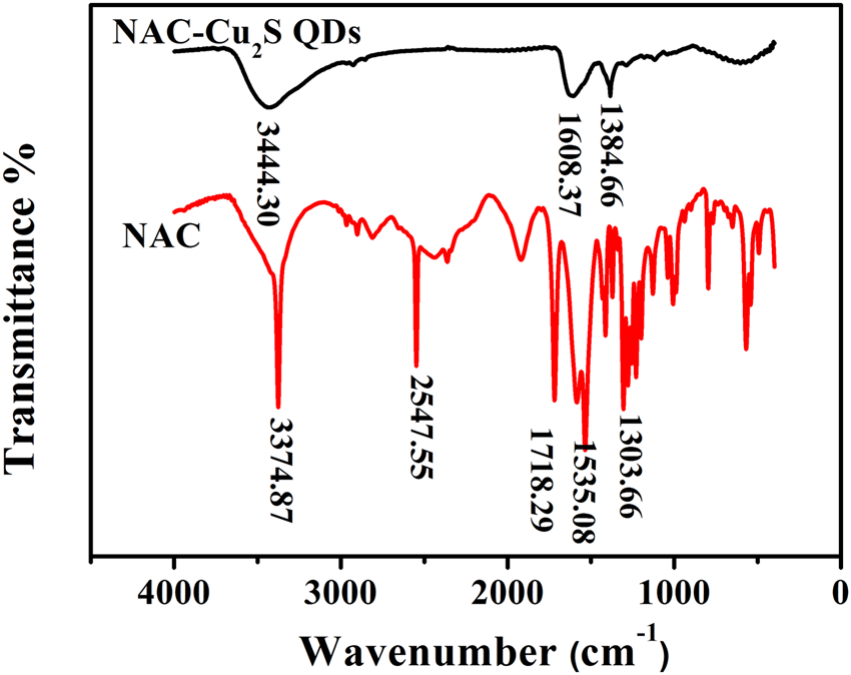
FTIR spectra of free NAC and the as-synthesized Cu_2_S QDs.

The FT-IR spectra of free NAC and NAC capped Cu_2_S are shown in Fig. 2. The broad bands around 3300–3600 cm^−1^ in the spectra may arise from the hydroxyl groups and H_2_O bound on the QDs surface. In the curve of NAC, the characteristic bands at 2547.55 cm^−1^ are attributed to S-H group^21^. Furthermore, the peaks at 1718.29 cm^−1^ and 1303.66 cm^−1^ are corresponding to carboxyl group, and the bands at 1190–1170 cm^−1^ are ascribed to C-N group, 1535.08 cm^−1^ is due to N-H stretching vibrations of -NH-^22^. The disappearance of the characteristic bands of S-H group in the curve of NAC capped Cu_2_S QDs indicates the successful bonding between the sulfur atom and the surface of Cu_2_S QDs. The shift of the C=O peak^23^ and N-H peak provides the evidence of electrostatic interaction between carboxyl groups and secondary amine of NAC and the Cu atom on the surface of the NAC capped Cu_2_S QDs.

**Figure 3:**
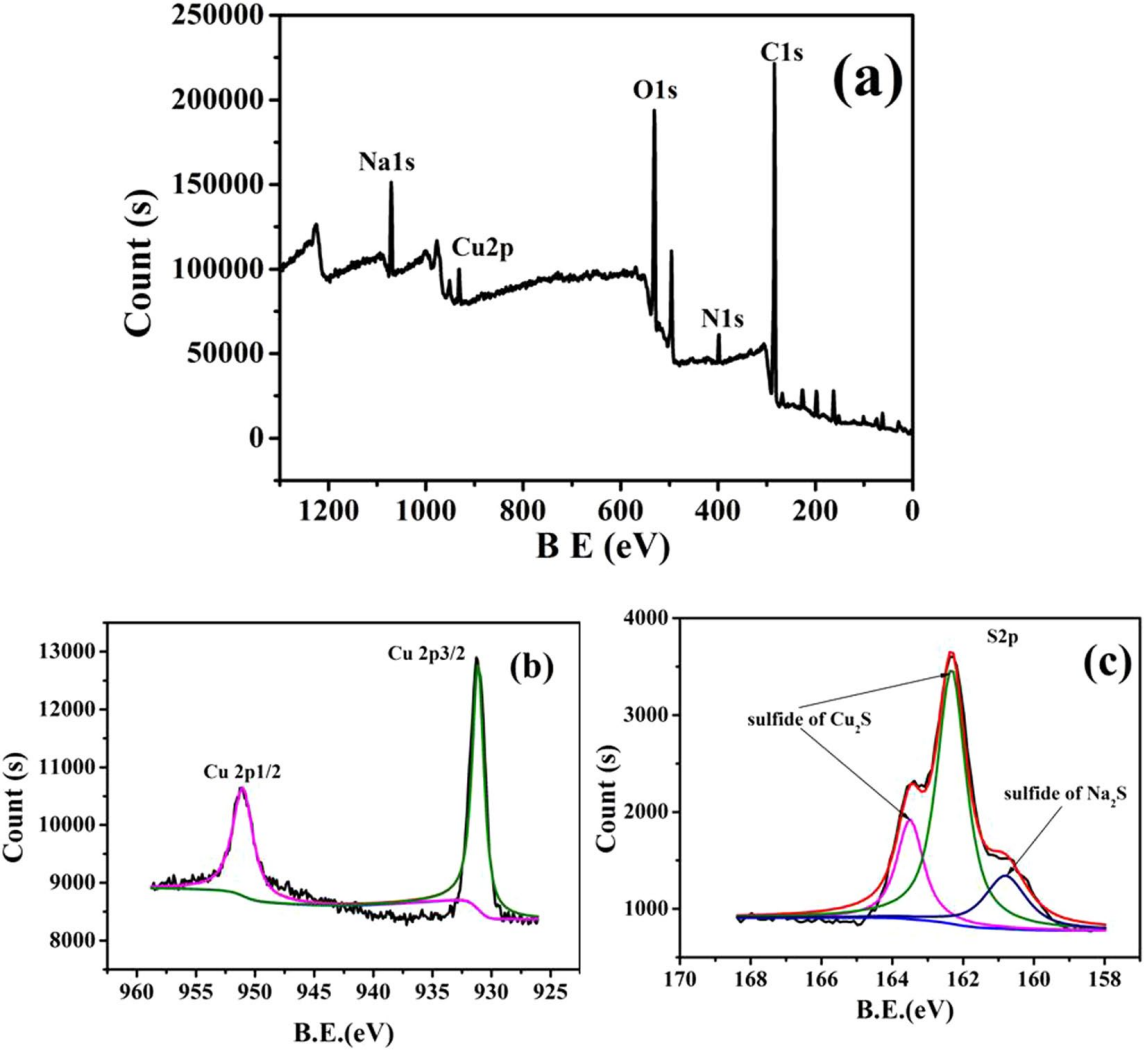
(**a**) XPS results of Cu_2_S QDs; (**b**) The amplified XPS of Cu 2p electrons; (**c**) The amplified XPS of S 2p electrons.

Evidence of elements composition and valence state of QDs can be obtained from the XPS analysis. As shown in Fig. 3a, five major peaks of Cu(2p), O(1 s), N(1 s), C(1 s), and S(2p) were found in XPS spectra of NAC capped Cu_2_S QDs. The binding energy position for Cu 2p_3/2_ and Cu 2p_1/2_ were located at 931.78 eV and 951.58 eV (Fig. 3b), the absence of satellite peaks indicates the absence of Cu^2+^ ^24^. Figure 3(c) shows the XPS spectra of the QDs in the region S 2p, peaks at 162.28 eV and 163.48 eV are identified as the S(2p_3/2_) and S(2p_1/2_), the difference in binding energy of the corresponding peaks is measured 1.2 eV and shows an agreement with published values of the S(2p) signal for Cu_2_S^25–27^. Peak at 160.78 eV is the S 2p signal contributed by residual Na_2_S^25^. No sulfur oxides (167–169 eV) were observed in samples which indicates that sulfur is in Cu_2_S form as S^−2^ species. The XPS results also reveal the formation of Cu_2_S with adsorbed Na_2_S species^26, 27^.

**Figure 4:**
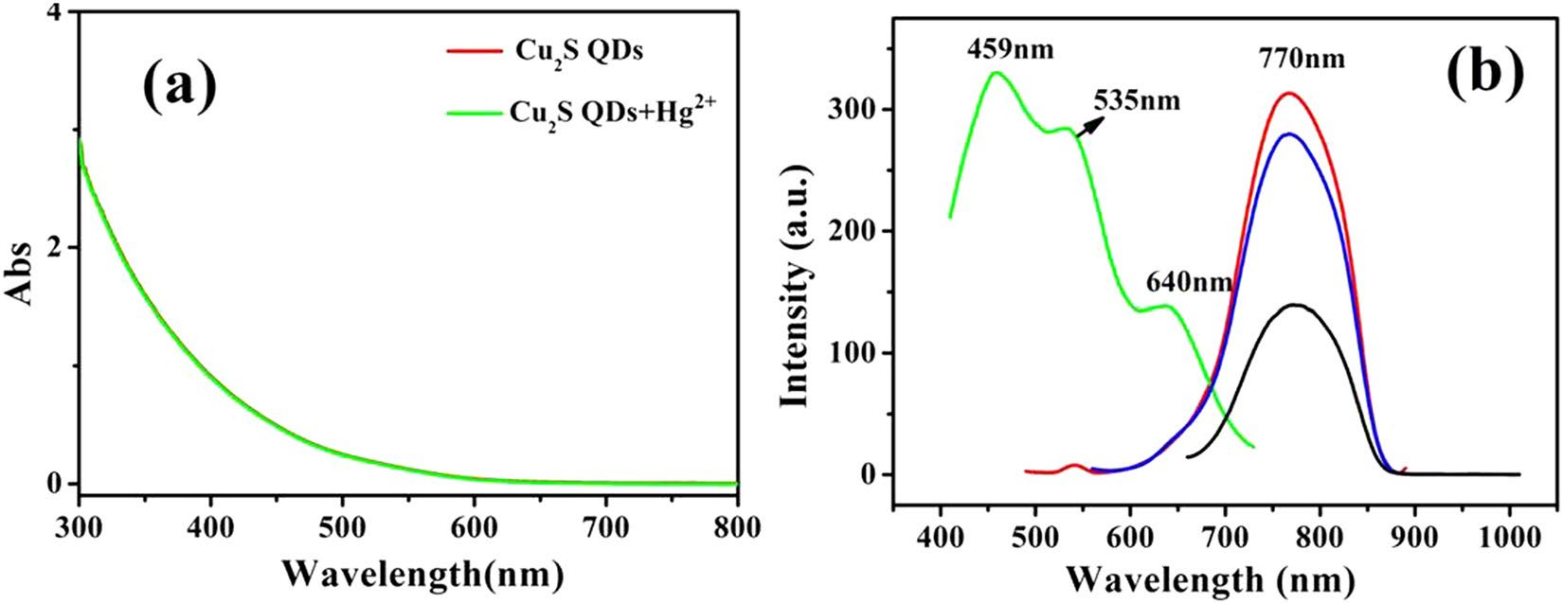
UV-visible absorption spectra (**a**) and Excitation spectrum (left) and PL spectra (right) (**b**) of Cu_2_S QDs.

Figure 4(a) is the absorption spectra of the QDs solution that shows a wide absorption from ultraviolet region up to near-infrared(NIR) band, which is similar with the reported of Cu_2_S nanocrystals preparation in a mixed solvent of dodecanethiol and oleic acid^8^. Recently, localized surface plasmon resonance (LSPR) is developed to elucidate the various stoichiometries in colloidal copper sulfide NCs. LSPR in the NIR absorption band was not observed in our experiments further indicates that the prepared Cu_2−x_S QDs is Cu_2_S^28^. We note that no change was observed in the shape and the intensity of the absorption spectra of Cu_2_S QDs after adding heavy metal ions (e.g. the green curve in Fig. 4(a), the concentration of Hg^2+^ is 2.67 × 10^−7^mol/L, Figs S5 and S6), which suggests the unchanged of the particle size of Cu_2_S QDs. The photoluminescence (PL) excitation and emission spectra of NAC capped Cu_2_S QDs are shown in Fig. 4(b). As can be seen from the spectra, only one emission peak was observed and located at 760–780 nm in the near infrared region corresponding to different excitation peaks. The maximum emission intensity of the functionalized Cu_2_S QDs was obtained at 770 nm with the excitation wavelength at 459 nm. The excitation spectrum shows three excitation peaks located at 459 nm, 535 nm and 640 nm, respectively, which are attributed to the quasi-continuous energy levels of NAC capped Cu_2_S QDs splitting into discrete energy levels. The only one luminescence band suggests the narrow size distribution of Cu_2_S QDs.

### Effects of different molar ratio on fluorescence intensity

**Figure 5:**
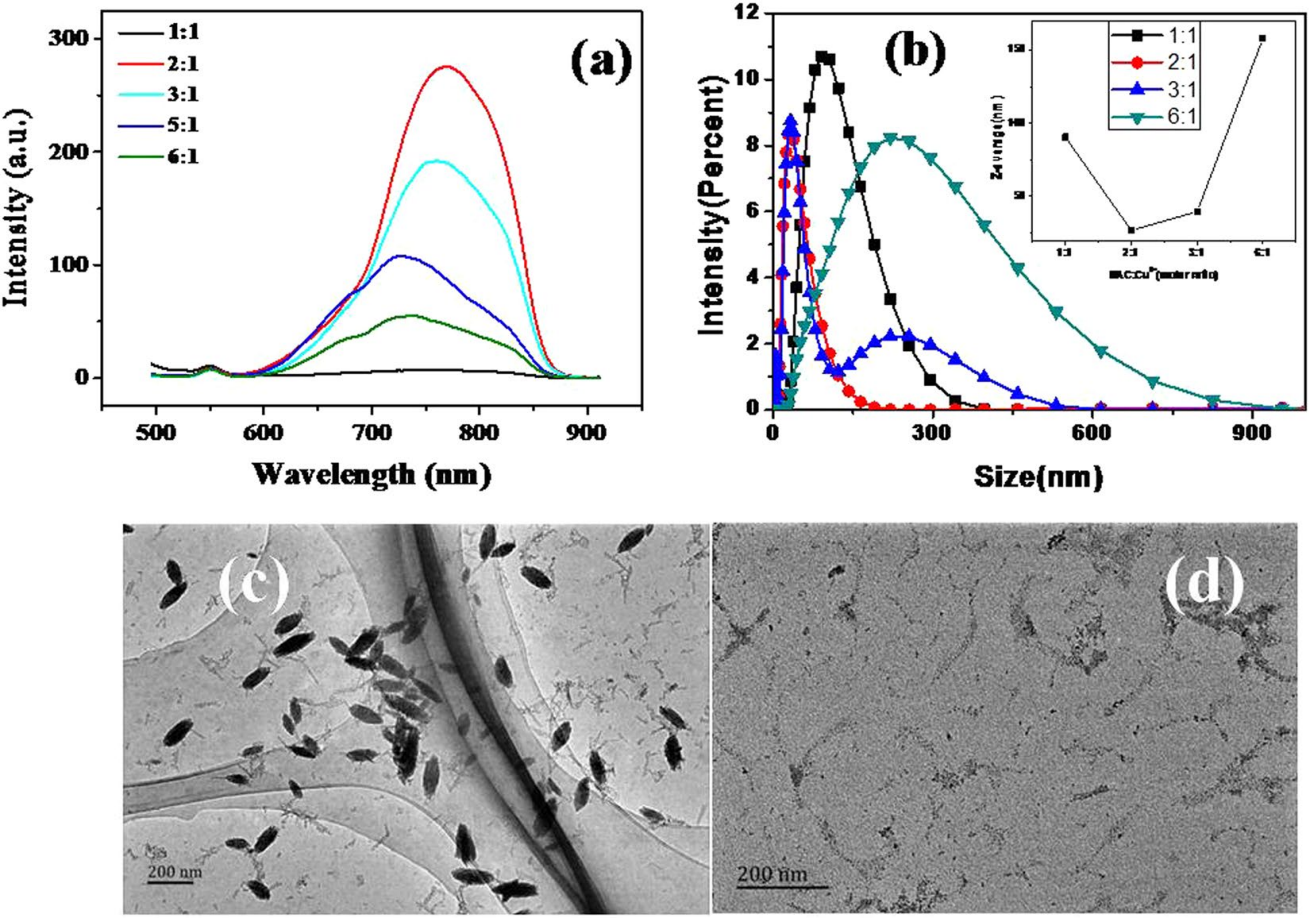
(**a**) Effects of NAC/Cu^2+^ molar ratio on the fluorescence intensity of Cu_2_S QDs; (**b**) Effects of NAC/Cu^2+^ molar ratio on the QDs size and size distribution of Cu_2_S QDs in solution; (**c**) and (**d**)TEM images of the Cu_2_S QDs synthesized with the molar ratio of NAC/Cu^2+^ = 1:1 and 6:1.

The optical properties of Cu_2_S QDs synthesized in different stabilizers are shown in Fig. S1, which can be seen that strong near-infrared(NIR) emitting Cu_2_S QDs were achieved only in NAC. The effect of different molar ratios of NAC/Cu^2+^ on the fluorescence spectra of NAC capped Cu_2_S QDs is showed in Fig. 5(a). It can be seen that the molar ratio of NAC/Cu^2+^  = 2:1 is optimal which has the highest emission intensity. The fluorescence intensity at the molar ratio of NAC/Cu^2+^ = 1:1 is lower than that of NAC/Cu^2+^ = 2:1, probably because the Cu_2_S QDs were not covered by NAC sufficiently. As a result, more clusters of QDs were generated due to the poor dispersion, and the emission intensity was reduced by non-radiative energy transfer. On the other hand, at the molar ratios of NAC/Cu^2+^ = 3:1, 4:1, 5:1 and 6:1, the higher concentration of NAC molecules in the suspension resulted in lower fluorescence intensity as well. The possible reason for this effect is that the high concentration of NAC in the suspension may increase the rate of molecular collision and promote the formation of the QDs clusters, the poor dispersion would introduce more non - radiative energy dissipation and reduce the fluorescence intensity of QDs^29^. In Fig. 5(a), we note that the fluorescence spectrum had a slight blue shift with the increase of the reactant ration of NAC/Cu^2+^, which indicates the changing size of QDs. In order to explain the spectral shift in the fluorescence emission, a particle size analysis and TEM observation were carried out on a particle size analyzer. Figure 5(b) depicts the variation of the Cu_2_S QDs size and size distribution with the variation ratios of NAC/Cu^2+^. The inserted in Fig. 5(b) shows that the Cu_2_S QDs size in the solution increased with the increase of the molar ratio of NAC/Cu^2+^ in a range from 2:1 to 6:1. The spectral shift and the QDs size change can be elucidated by the interaction of NAC and Cu^2+^. Figure 5(c) and (d) show the TEM images of the Cu_2_S QDs prepared in the molar ratio of NAC/Cu^2+^ = 1:1 and 6:1, the presence of some large particles is observed as it was detected from particle size analyzer and confirmed the size change of the QDs. Cu^2+^ can form a NAC-Cu^+^ complex by covalent bond with dehydrogenized –SH group from NAC in the solution. The number of covalent bond of the Cu^+^ and NAC increases with the increase of the molar ratio of NAC/Cu^2+^, which might affect the nucleation rate of Cu_2_S QDs after adding S^2−^ and results in the variation of QDs size and the blue shift of fluorescence spectrum.

**Figure 6:**
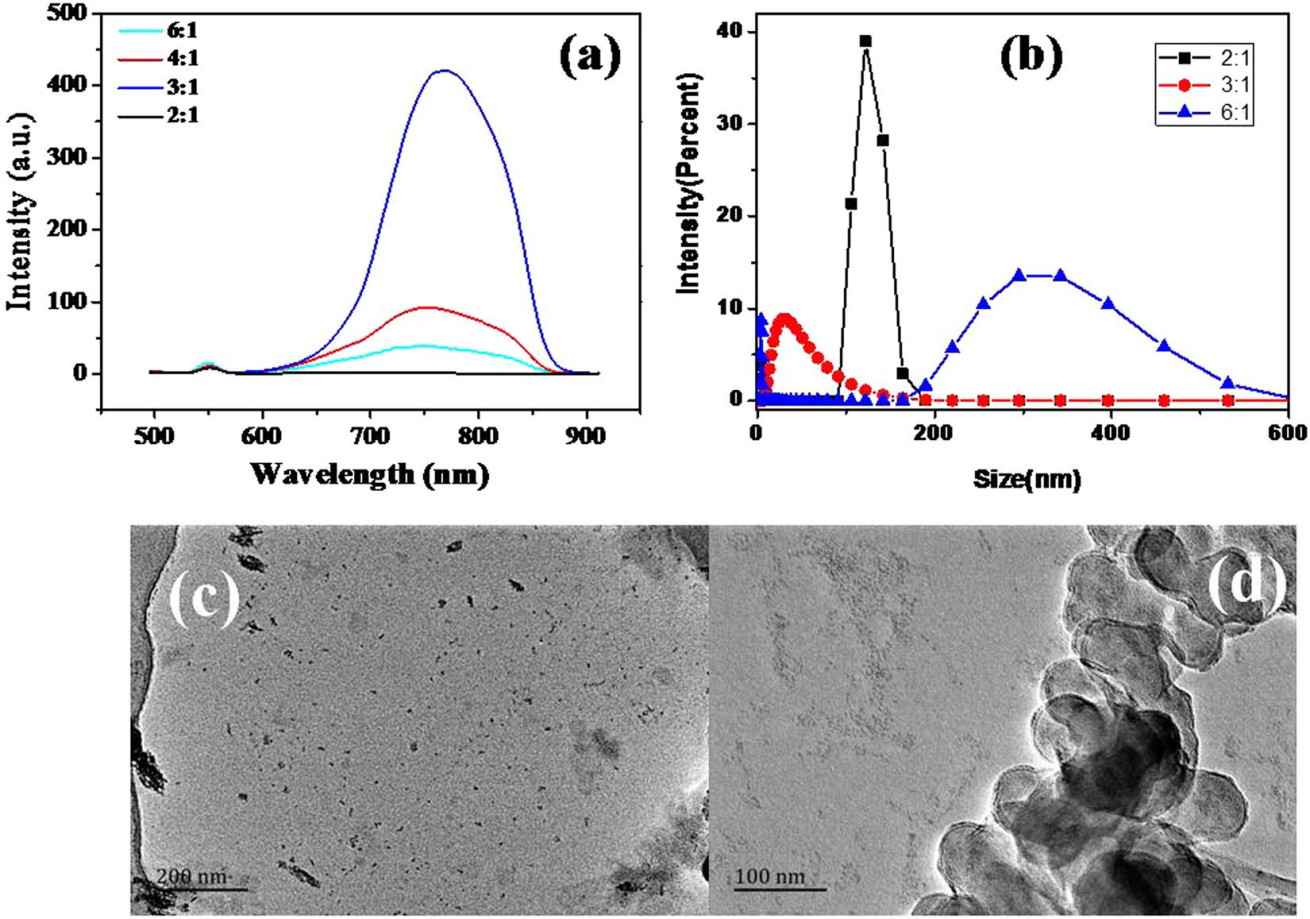
(**a**) Effects of Cu^2+^/S^2−^ molar ratio on the fluorescence intensity of Cu_2_S QDs; (**b**) Effects of Cu^2+^/S^2−^ molar ratio on the Cu_2_S QDs size and size distribution in solution. (**c**) and (**d**) TEM images of the Cu_2_S QDs synthesized with the molar ratio of Cu^2+^/S^2−^ = 2:1 and 6:1.

Figure 6(a) depicts the PL spectra of NAC capped Cu_2_S QDs with different molar ratios of Cu^2+^/S^2−^ under the excitation wavelength of 466 nm. It is found that the PL emission intensity significantly increased with the increased of Cu^2+^/S^2−^ ratio, up to a maximum when the ratio is 3:1. However, the fluorescence intensity of Cu_2_S QDs gradually decreased with the further increase of Cu^2+^/S^2−^ ratio. The result of particle size analysis shows that the QDs size varied with the variation of molar ratios of Cu^2+^/S^2−^, the smallest QDs size was achieved at the Cu^2+^/S^2−^ ratio of 3:1, as shown in Fig. 6(b). Figure 6(c) and (d) indicate the TEM images of the Cu_2_S QDs prepared at the molar ratio of Cu^2+^/S^2−^ = 2:1 and 6:1, the presence of some large particles is observed as it was detected from particle size analyzer.

### Effects of different temperature and pH value of the original solution

**Figure 7:**
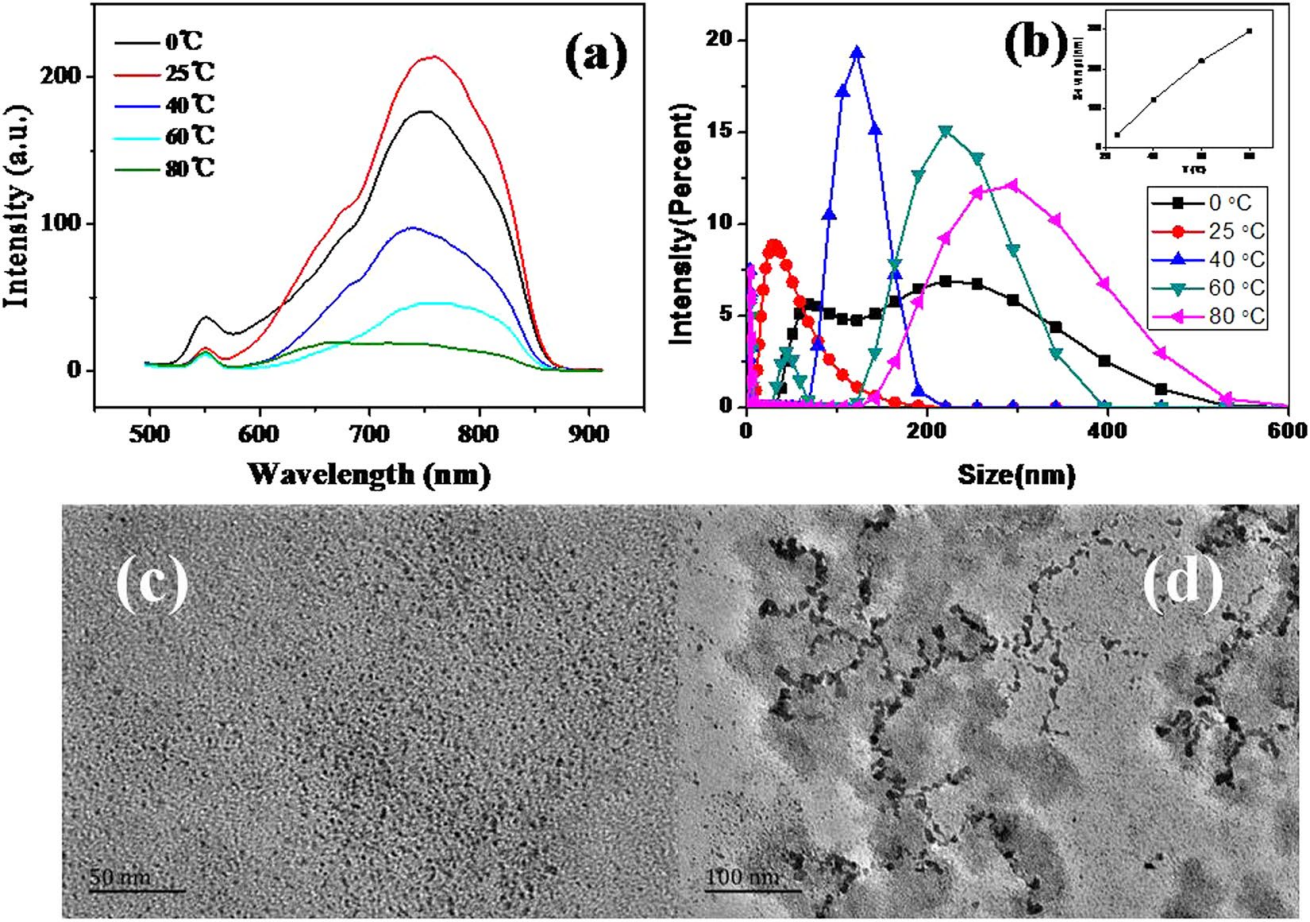
(**a**) Effects of temperature on the fluorescence intensity of Cu_2_S QDs; (**b**) Effects of temperature on the Cu_2_S QDs size and size distribution in solution; (**c**) and (**d**) TEM images of the Cu_2_S QDs synthesized at the temperature of 40 °C and 80 °C.

The effect of reaction temperatures was investigated on the fluorescence spectra of Cu_2_S QDs. As shown in Fig. 7(a), the maximum fluorescence intensity was obtained at 25 °C when the temperature varied from 0 °C to 80 °C. The shift towards lower wavelength and the fluorescence quenching with the increased temperature were observed. Yongbo Wang *et al*.^30^ reported that the higher temperature leaded to the rapid nucleation and growth process, producing QDs with large size and a lot of surface defects and thus causing a reduction in the fluorescence intensity of QDs. Temperature effect competition between radiative and nonradiative transitions have been found by Mohamed and Pendyala *et al*.^31, 32^. Figure 7(b) shows the variation of the Cu_2_S QDs size and size distribution with the temperature. The inserted in Fig. 7(b) shows that the QDs size increased with the increase of the temperature in the range from 25 °C to 80 °C, which is according to the previous reports^30^. Figure 7(c) and (d) show the TEM images of the Cu_2_S QDs synthesized at the temperature of 40 °C and 80 °C, which confirms that the average size of the QDs increased with the increase temperature. The presence of some large particles in Fig. 7(d) is observed as it was detected from particle size analyzer.

**Figure 8:**
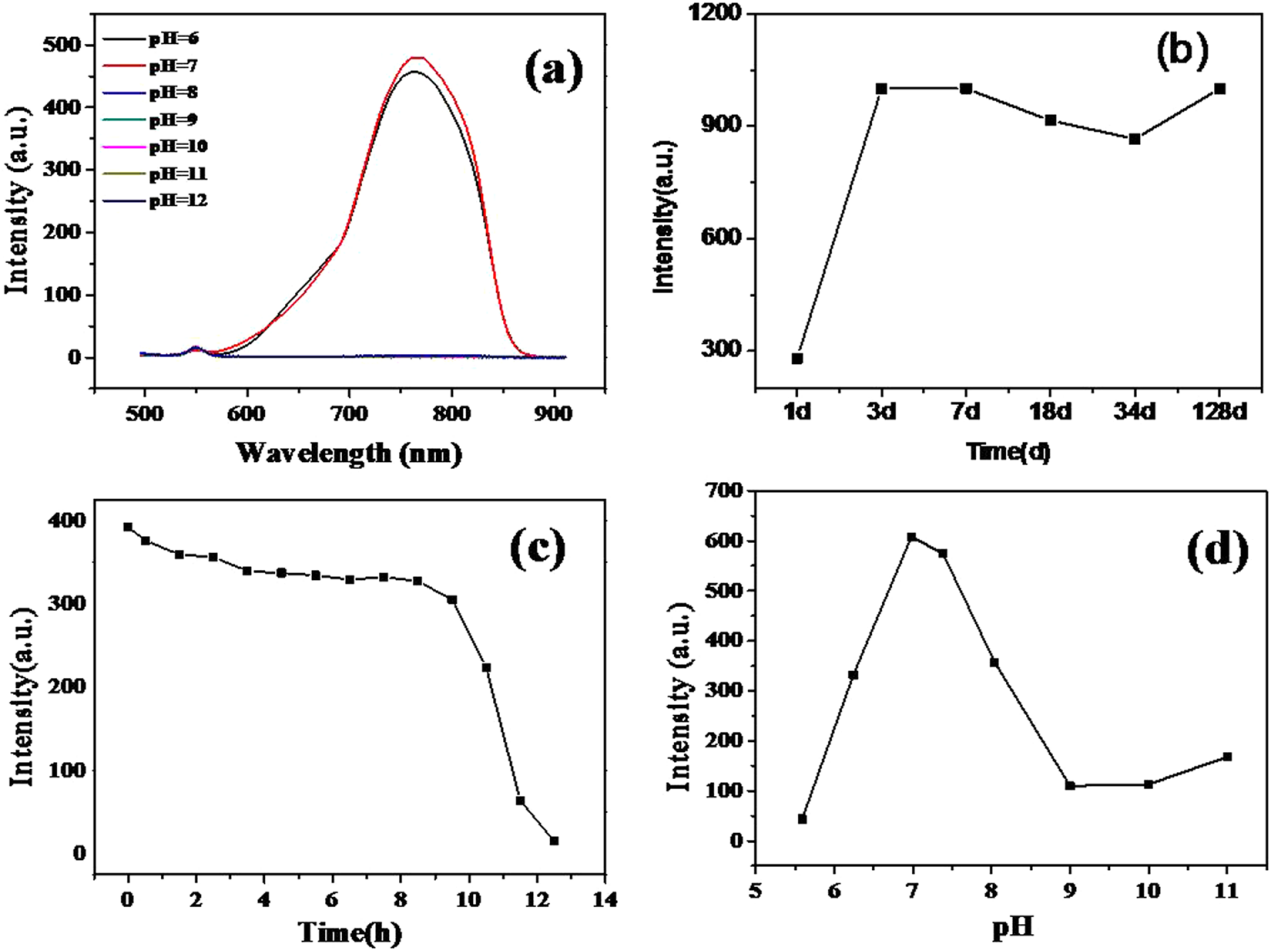
(**a**) Effects of pH values on the fluorescence intensity of Cu_2_S QDs; (**b**) Colloidal stability of the as-prepared Cu_2_S QDs; (**c**) Time-dependent fluorescence response of the Cu_2_S QDs to be exposed to the air; (**d**) Fluorescence intensity of the Cu_2_S QDs in PBS at different pH values.

To study the effect of pH on the fluorescence spectra, NAC capped Cu_2_S QDs were synthesized in different pH values. As shown in Fig. 8(a), the fluorescence intensity increased with the increase of pH values and reached the optimum at pH = 7, then decayed with the further increase of pH values, no obvious fluorescence emission peak was found when pH > 9. The bond energy can be used to explain the effect of pH value on Cu_2_S QDs fluorescence intensity^33^. As the pH value of the solution varied from 6 to 12, the covalent bond between Cu^+^ and dehydrogenized –SH group from NAC is dramatically strengthened^22, 25^. When the bond energy of –SH group and Cu^+^ is larger than that of Cu^+^ and S^2−^, it will hinder the formation of Cu-S bond. As pH < 7, the turbid solution indicates the formation of NAC-Cu^+^ complexes precipitation. Bond energy of –SH group and Cu^+^ is smaller than that of Cu^+^ and S^2−^ while pH = 7, the –SH group from NAC is attached to Cu^+^, forming NAC- Cu^+^ complexes and wrapped on the surface of Cu_2_S QDs, which dramatically increases the fluorescence intensity^34^. Consequently, the pH value of 7 is recommended to use in our experiments.

### Stability of Cu_2_S QDs

In the dark sealed conditions, the Cu_2_S QDs solution can be kept at room temperature for more than four months as shown in Fig. 8(b). The fluorescence intensity of the as-prepared QDs was significantly enhanced at the second day, indicating a thick shell of NAC-Cu^+^ complexes was formed on the QDs’ surface, thus dramatically improved the fluorescence intensity^33^. Figure 8(c) shows the plot of the fluorescence intensity of Cu_2_S QDs exposed to the air vs. the exposure time. As can be seen from Fig. 8(c), there is a platform from 3 h to 9 h, which indicates that Cu_2_S QDs only can be kept in the air for 6 h. The fluorescence intensity of Cu_2_S QDs quickly decreased when kept for longer than 9 h, the fluorescence quenching of Cu_2_S QDs in the air may be due to the oxidation^8^.

### Effect of different pH values of buffer solution

The pH value effect of the PBS buffer solution towards the fluorescence of Cu_2_S QDs was studied in order to select the optimal conditions for detecting heavy metal ions. The fluorescence intensity under different pH values in a range between 5.0 and 11.0 is shown in Fig. 8(d). The fluorescence intensity of Cu_2_S QDs increased with pH value and reaches maximum at pH 6.98 in PBS buffered aqueous medium, then decreases with pH value further increases. Therefore, the ideal pH level of PBS buffer solution is 6.98 for the determination of heavy metal ions.

### The influence of different ions on the Cu_2_S QDs fluorescence intensity

**Figure 9:**
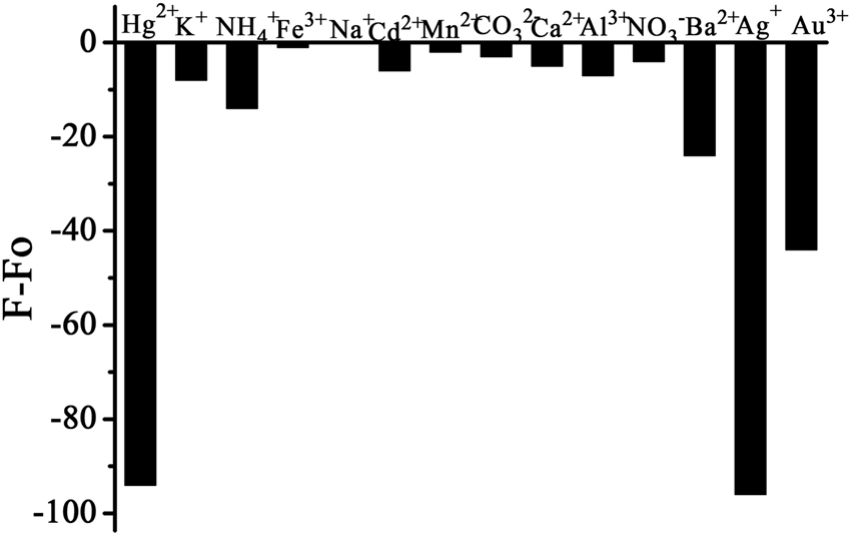
The quenching effect of different ions on the fluorescence intensity of the Cu_2_S QDs.

In this work, a response of fluorescence spectra to various ions including Hg^2+^, Ag^+^, Au^3+^, Na^+^, K^+^, NH_4_^+^, Fe^3+^, Cd^2+^, Mn^2+^, Ca^2+^, Al^3+^, Ba^2+^, CO_3_^2−^ and NO_3_^−^ ions at concentration of 2 × 10^−5^ mol/L(except for Hg^2+^ of 5.3 × 10^−7^ mol/L, Ag^+^ of 8 × 10^−7^ mol/L, Au^3+^ of 2.43 × 10^−5^ mol/L) in NAC capped Cu_2_S QDs(4 × 10^−4^ mol/L) solution was studied in pH 6.98, PBS. The fluorescence quenching degree (ΔF) of Cu_2_S QDs to different ions is depicted in Fig. 9. We found that the fluorescence of Cu_2_S QDs was significantly quenched by heavy metal ions of Hg^2+^, Ag^+^, Au^3+^ without any emission band shift (as shown in Figs S2, S3 and S4), slightly quenched by ions of Ba^2+^ and NH_4_^+^. Other ions such as Na^+^, K^+^, Fe^3+^, Cd^2+^, Mn^2+^, Ca^2+^, Al^3+^, CO_3_^2−^ and NO_3_^−^ displayed no quenching response to Cu_2_S QDs. So we can infer that the prepared Cu_2_S QDs have high detection sensitivity and selectivity for Hg^2+^, Ag^+^ and Au^3+^ ions.

### Sensing performance of Cu_2_S QDs for Hg^2+^, Ag^+^ and Au^3+^ ions

**Figure 10:**
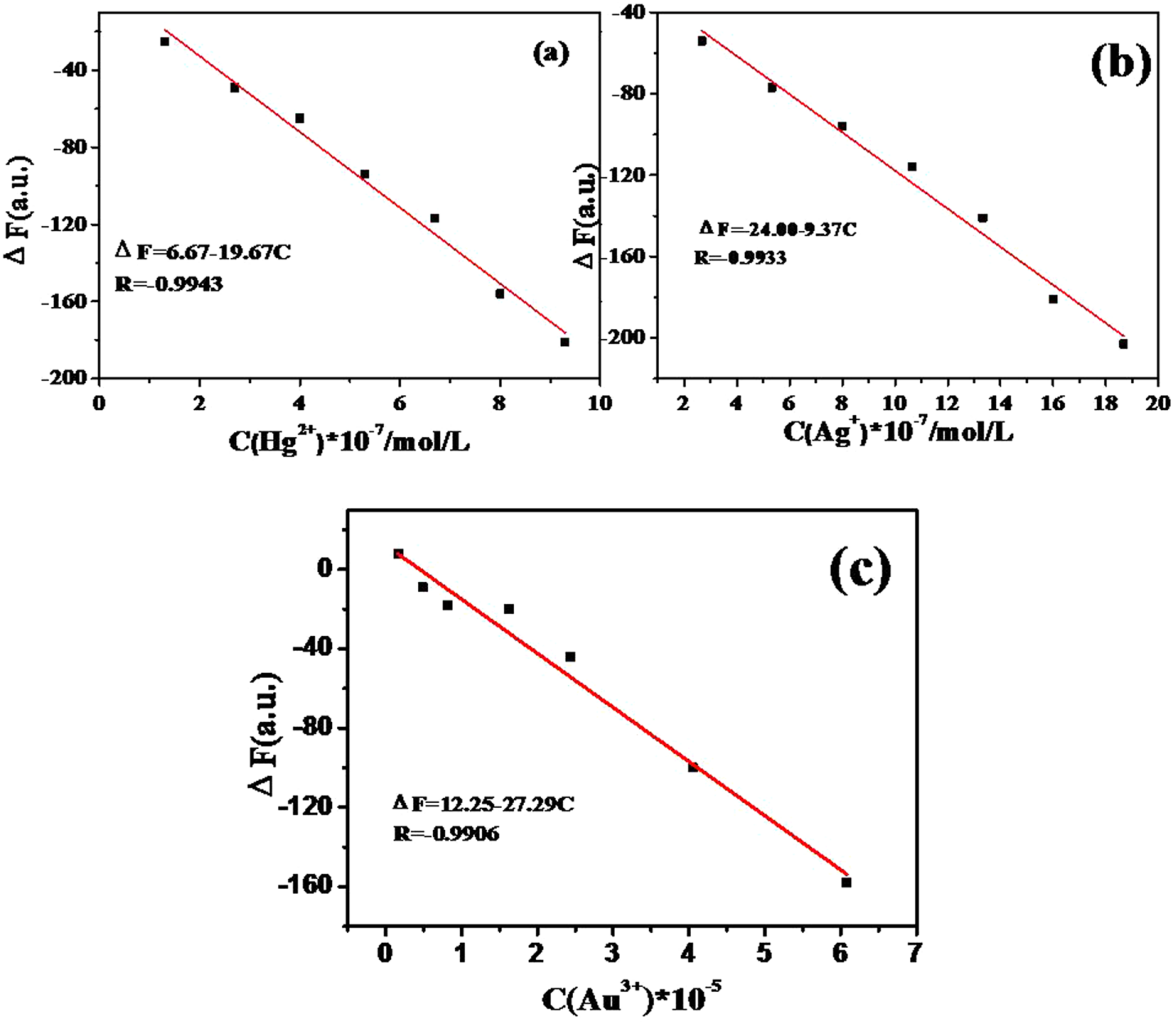
Calibration curves of the degree of fluorescence quenching (ΔF) of NAC capped Cu_2_S QDs versus (**a**)Hg^2+^, (**b**)Ag^+^, (**c**)Au^3+^ ions concentration.

Under the optimum experimental conditions, the effective quenching of the fluorescence of Cu_2_S QDs response to different concentrations of Hg^2+^, Ag^+^ and Au^3+^ ions was investigated respectively. The fluorescence intensity of NAC capped Cu_2_S QDs at 770 nm decreased significantly with the increase of concentration of Hg^2+^, Ag^+^ and Au^3+^, respectively. The quenching fluorescence is proportional to the concentration of Hg^2+^, Ag^+^ and Au^3+^, respectively. Figure 10 shows that there is a good linear relationship between the ΔF of NAC capped Cu_2_S QDs and the concentration of the three heavy metal ions, with an R^2^ value close to unity. The linear response of Cu_2_S QDs emission proportional to the concentration of Hg^2+^ ion ranging from 1.33 × 10^−7^ to 9.33 × 10^−7^ mol/L with a detection limit of 3.99 × 10^−8^ mol/L (Fig. 10(a)), 2.67 × 10^−7^ to 1.89 × 10^−6^ mol/L with the detection limit of 1.33 × 10^−8^ mol/L for Ag^+^ ion (Fig. 10(b)), and 1.62 × 10^−6^ to 6.08 × 10^−5^ mol/L with a detection limit of 1.62 × 10^−7^ mol/L for Au^3+^ ion (Fig. 10(c)).

### Quenching mechanism of Hg^2+^, Ag^+^ and Au^3+^ ions on fluorescence of Cu_2_S QDs

To date, several quenching mechanisms have been proposed to explain how metal ions quench the fluorescence of functionalized QDs. Inner filter effect, non radiative recombination pathway, electron transfer process, aggregate-induced quenching, metal ion displacement and ion binding interaction are the possible mechanisms to explain the quenching phenomena^8, 23^. In our study, we speculated Hg^2+^, Ag^+^ and Au^3+^ could quench the fluorescence of Cu_2_S QDs mainly via electron transfer with cation exchange and aggregate-induced quenching.

**Figure 11:**
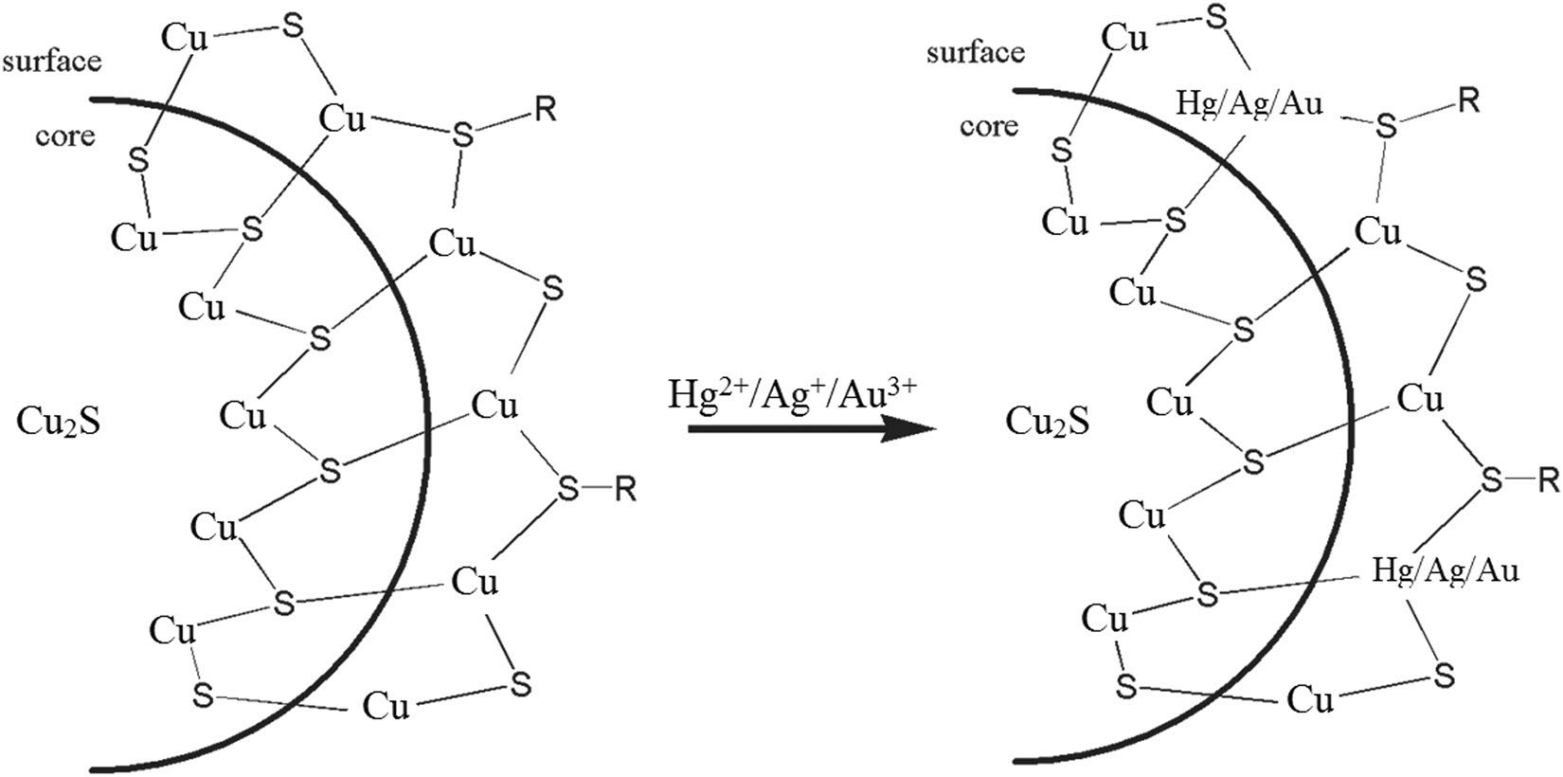
Illustration of the surface of NAC capped Cu_2_S QDs and their interaction with Hg^2+^, Ag^+^, Au^3+^ ions. R represents the moiety of NAC.

According to Hard-Soft-Acid-Base (HSAB) theory, the Lewis acid-base can be classified into soft acid, hard acid, soft base, hard base and medium acid-base^35^. Cu^+^, Hg^2+^, Ag^+^ and Au^3+^ are soft acids. RS^−^ belongs to soft base. As referred to above, Cu^+^ and dehydrogenized –SH group from NAC was formed a covalent bond on the surface of NAC-Cu_2_S QDs. After the addition of Hg^2+^, Ag^+^ and Au^3+^ ions respectively, the covalent bond between Hg^2+^, Ag^+^, Au^3+^ and thiols at the surface of NAC-Cu_2_S QDs were also produced. The covalent bond strength can be characterized by their corresponding metal –sulfide bond strength with their respective Ksp value. The Ksp value of HgS(1.6 × 10^−52^), Ag_2_S(6.0 × 10^−50^), Au_2_S_3_(According to literature^36^, not higher than 10^−50^) is much lower than that of Cu_2_S(3.0 × 10^−48^). The fluorescence of QDs is sensitive to their surface states, some changes of the surface charges of QDs would change their photophysical properties. Previous reports on some metal cations, such as Hg^2+^, Cu^2+^ and Cr^3+^ ions can quench fluorescence of QDs through interacting with the capping layer of QDs^37^. Figure 11 depicts the schematic illustration of the surface of NAC capped Cu_2_S QDs and its interaction with metal ions. The thick shell of NAC-Cu^+^ complexes on the surface of Cu_2_S QDs could be displaced by NAC-Hg^2+^ complexes, NAC-Ag^+^ complexes and NAC-Au^3+^ complexes, which resulted in imperfections on the Cu_2_S QDs surface and facilitate non-radiative e^−^/h^+^ recombination through an effective electron transfer process^38, 39^. Another possible explanation is aggregate - induced quenching. Since the surface of Cu_2_S QDs is attached with a lot of carboxylate groups in NAC. Hg^2+^, Ag^+^ and Au^3+^ might exhibit a certain affinity to carboxylate groups on the surface of Cu_2_S QDs and induce the Cu_2_S QDs aggregated to some extent. As a consequence, the fluorescence of Cu_2_S QDs gets quenched with Hg^2+^, Ag^+^ or Au^3+^ ion adding into the Cu_2_S QDs solution gradually.

**Figure 12:**
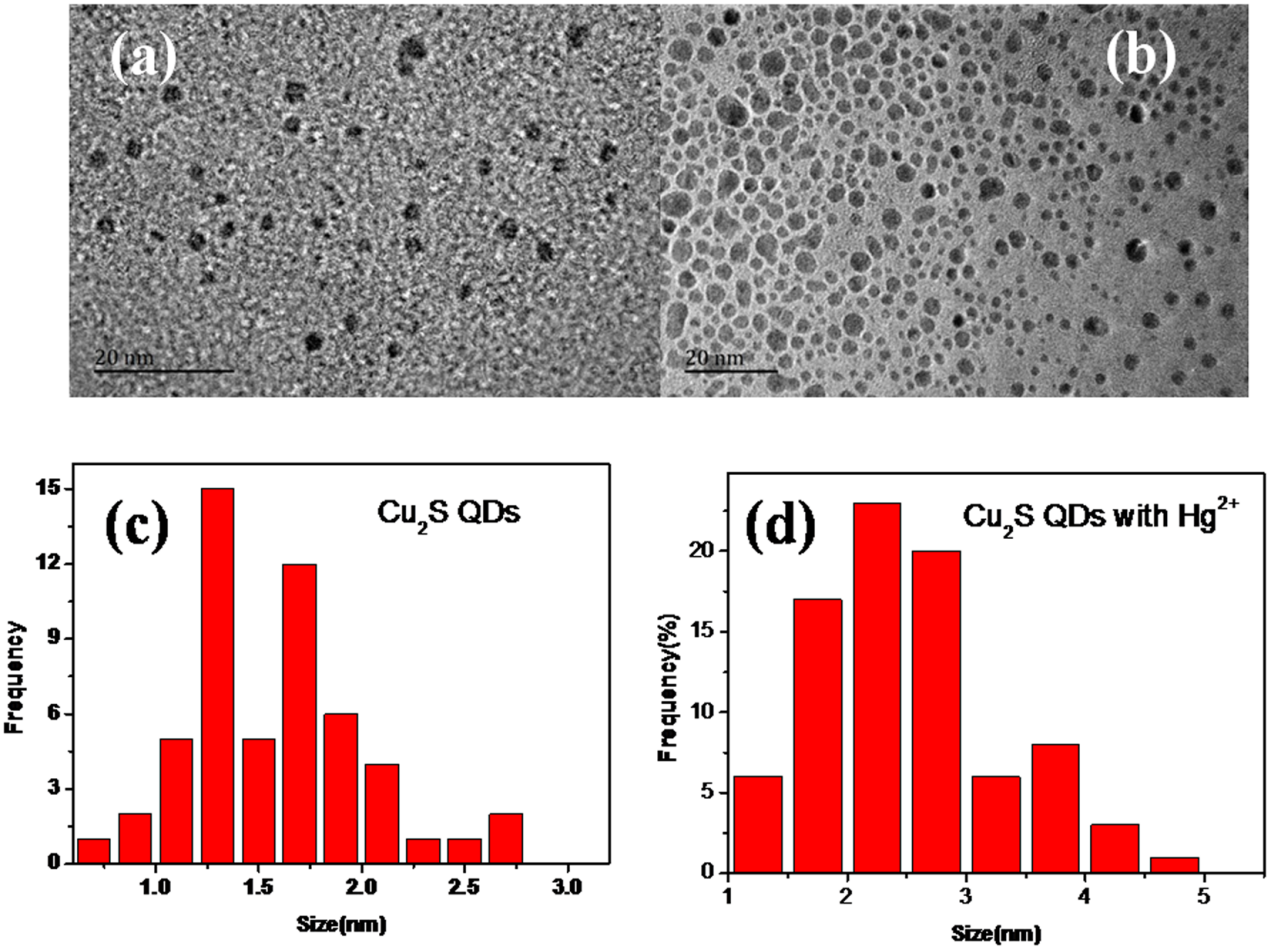
TEM images of the as-synthesized Cu_2_S QDs in PBS buffer solution (pH = 6.98) (**a**) and the presence of Hg^2+^ (1 × 10^–7^ mol/L) (**b**); size and size distribution of the as-synthesized Cu_2_S QDs in PBS buffer solution (pH = 6.98) (**c**) and QDs in the presence of Hg^2+^ (1 × 10^−7^ mol/L) (**d**).

As depicted in Fig. 4(a), no shift was observed in the absorption spectra of Cu_2_S QDs with addition of Hg^2+^, Ag^+^ or Au^3+^, indicating the unchanged QDs size. In addition, there was also no spectral shift in fluorescence spectra although the fluorescence intensity of Cu_2_S QDs was quenched in the presence of Hg, Ag^+^ and Au^3+^ ions respectively. Figure 12 indicates the TEM images of the as-synthesized Cu_2_S QDs in PBS buffer solution (pH = 6.98) (Fig. 12a) and containing 1 × 10^−7^ mol/L Hg^2+^ ions(Fig. 12b). Compared with Fig. 12(a) and (b), both of the Cu_2_S QDs are of around 1–5 nm size and have a narrow size distribution and good dispersibility, as shown in Fig. 12(c) and (d). The presence of large particles was not observed, which excludes the aggregate - induced quenching and confirms that the fluorescence quenching of the Cu_2_S QDs in the presence of heavy metal ions might be mainly induced by electron transfer process. The detailed mechanisms still need to be further studied and the relevant work is in progress.

## Conclusion

In conclusion, stable water-soluble NAC capped Cu_2_S QDs with near-infrared emission were successfully synthesized via a facile strategy. The fluorescence properties of the QDs were optimized by adjusting the experimental variables and the maximum fluorescence intensity of the as-prepared QDs were obtained when NAC/Cu^2+^ and Cu^2+^/S^2−^ ratios of 2 and 3, respectively, pH value at 7 and reaction temperature at 25 °C. It was also demonstrated that the fluorescence of the as-prepared NAC capped Cu_2_S QDs could be quenched by Hg^2+^, Ag^+^ and Au^3+^ ions with high sensitivity and selectivity. The mechanism of fluorescence quenching in the presence of Hg^2+^, Ag^+^ and Au^3+^ ions might be attributed to the non-radiative e^−^/h^+^ recombination on the NAC capping layer. The as-prepared Cu_2_S QDs have potential applications in the detection of other metal ions and many other fields.

## Electronic supplementary material

supplementary Info
