# Supplementary material for: Aqueous synthesis of functionalized copper sulfide quantum dots as near-infrared luminescent probes for detection of Hg2+, Ag+ and Au3+
Source: Sci Rep. 2017 Sep 13;7:11451. doi: 10.1038/s41598-017-10904-y (PMC5597581; doi:10.1038/s41598-017-10904-y)
Supplement: Supplementary file 1 — supplementary Info [file 41598_2017_10904_MOESM1_ESM.doc]

**Aqueous synthesis of functionalized copper sulfide quantum dots as near-infrared luminescent probes for detection of Hg^2+^, Ag^+^ and Au^3+^**

**Weilin Du^1^, Lei Liao^2^, Li Yang^1^, Aimiao Qin^1^*, Aihui Liang*^3^**

^1^Key Lab New Processing Technology for Nonferrous Metals & Materials Ministry of Education, Guangxi Key Laboratory in Universities of Clean Metallurgy and Comprehensive Utilization for Non-ferrous Metals Resources, College of Materials science & engineering, Guilin University of Technology, Guilin, China

^2^College of Environment science & engineering, Guilin University of Technology, Guilin, China

^3^College of Environment & Resource, Guangxi Normal University, Guilin, China

*corresponding Authors: 592491245@qq.com, hliang2008@163.com

**KEYWORDS: Cu_2_S QDs; aqueous synthesis; near-infrared emission; Quantitative analysis; Hg(II), Ag(I) and Au(III).**

**Figure S1 The PL spectra of room temperature synthesis of Cu_2_S QDs with different surface modification**

**Figure S2 (a) The PL spectra of NAC capped Cu_2_S QDs quenched by Hg^2+^ ion; (b) The linear relation between** **the degree of fluorescence quenching of NAC-Cu_2_S QDs and the Hg^2+^ concentration**

**Figure S3 (a) PL spectra of NAC capped Cu_2_S QDs quenched by Ag^+^ ion; (b) The linear relation between the degree of fluorescence quenching of NAC-Cu_2_S QDs and the Ag^+^ concentration**

**Figure S4 (a) PL spectra of NAC capped Cu_2_S QDs quenched by Au^3+^ ion; (b) The linear relation between the degree of fluorescence quenching of NAC-Cu_2_S QDs and the Au^3+^ concentration**

**Figure S5 UV-visible absorption spectra of NAC capped Cu_2_S QDs (4×10^-4^ mol/L)and QDs in the presence of Ag^+^(8×10^-7^ mol/L )**

**Figure S6 UV-visible absorption spectra of NAC capped Cu_2_S QDs (4×10^-4^ mol/L) and QDs in the presence of Au^3+^(2.43×10^-5^ mol/L)**
